# Unmasking hidden metastasis: A case report of gastrointestinal metastasis detected a decade after breast cancer diagnosis

**DOI:** 10.1097/MD.0000000000043893

**Published:** 2025-08-08

**Authors:** Jie Peng, Ye Liu, Yiheng Zhang, Haiyuan Yang, Guoxin Sun, Lichao Zhu, Yating Zhao

**Affiliations:** a Breast Disease Treatment Center, Affiliated Hospital of North China University of Science and Technology, Tangshan, Hebei Province, China.

**Keywords:** breast cancer, breast surgery, gastrointestinal metastasis of breast cancer

## Abstract

**Rationale:**

Breast cancer is among the most common malignancies in women, and metastasis significantly impacts survival and quality of life. Although many patients remain disease-free in the early years after surgery and adjuvant therapy, occult metastasis remains a major clinical concern. While bone, lung, and liver are common metastatic sites, gastrointestinal involvement is rare and often overlooked. Recent evidence suggests that late-stage occult metastasis can develop even after long disease-free intervals. Early detection is vital for optimizing treatment and improving prognosis. Therefore, gastrointestinal symptoms such as abdominal pain, altered bowel habits, or unexplained weight loss in breast cancer patients warrant prompt imaging and endoscopic evaluation.

**Patient concerns:**

This case report describes a 49-year-old woman with invasive lobular breast carcinoma who, after nearly 11 years of recurrence-free survival, presented with constipation and weight loss. Imaging and endoscopy revealed gastric and rectal metastases, underscoring the risk of occult metastasis in breast cancer. This case highlights the importance of considering age and treatment history when developing personalized management strategies and emphasizes early detection and intervention to improve outcomes and quality of life.

**Diagnoses:**

Computed tomography revealed thickening of the gastric antrum and rectal wall, resulting in luminal narrowing. A comprehensive flexible colonoscopy was performed, and biopsy of the colonic tissue for pathological examination indicated metastatic tumors in the rectal mucosa originating from breast cancer as well as metastatic lobular carcinoma in the stomach.

**Interventions:**

The patient is currently receiving a chemotherapy regimen of gemcitabine 1.4 g and carboplatin (500 mg).

**Outcomes:**

The patient is currently in the chemotherapy phase, and close monitoring of the condition is being conducted during treatment.

**Lessons:**

For patients with a history of breast cancer, the emergence of new gastrointestinal symptoms or a diagnosis of primary gastric or colorectal cancer should raise suspicion metastatic breast cancer. During the biopsy process, a comprehensive evaluation of histopathological and immunohistochemical analyses is necessary, and these results should be compared with those of primary breast cancer to ensure an accurate diagnosis.

## 1. Introduction

Breast cancer is the most frequently diagnosed malignancy in women. Globally, approximately 16.7% of cancer-related deaths in women are attributed to breast cancer.^[1,2]^ Breast cancer commonly metastasizes to the liver, bones, lungs, brain, and lymph nodes.^[3]^ Studies have reported that gastrointestinal metastases occur in approximately 2% to 18% of breast cancer patients, typically several years after diagnosis. Among these sites, the stomach is the most commonly affected, followed by the colon and rectum.^[4,5]^ Invasive lobular carcinoma (ILC), accounting for 10% to 20% of breast tumors, is particularly prone to metastasis due to the loss of intercellular adhesion molecules.^[6]^ Borst et al reported that ILC more frequently metastasizes to the gastrointestinal tract, gynecologic organs, and peritoneum compared to invasive ductal carcinoma. Among these sites, the stomach is the most frequent site of metastasis, followed by the small intestine, while involvement of the colon and rectum is relatively uncommon.^[7]^

## 2. Case report

**Figure 1.**
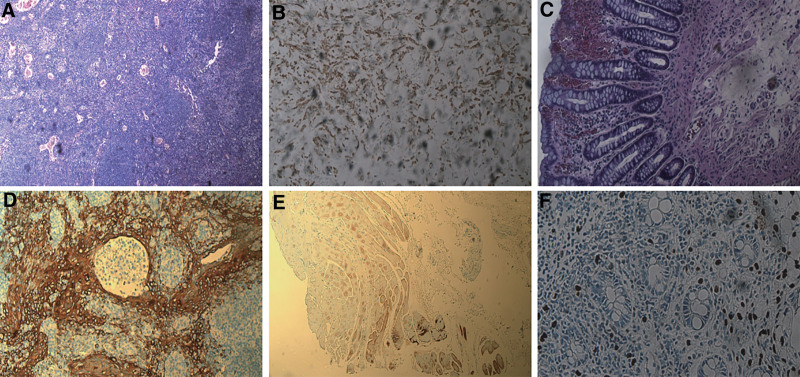
Immunohistochemical results. (A) The right breast shows fibrotic cancerous tissue with scattered lymphocytes, presenting an infiltrative growth pattern. (B) Staining revealed a branching, network-like structure, reflecting the infiltration and connectivity patterns of cancer cells. (C) Rectal tissue exhibits glandular structures resembling those of breast tissue, suggesting ectopic glands. Cell nuclei show variability and irregularity, with an increased nuclear-to-cytoplasmic ratio, indicating tumor cell heterogeneity. (D) Brown-stained cells are located around glandular structures, and the area contains epithelial cells with high expression of cytokeratin, reflecting the characteristics of adenocarcinoma. The brown-stained cells indicated positive cytokeratin staining, consistent with the high cytokeratin expression observed in breast cancer tumor cells. (E) The area of high proliferation showed significant Ki-67 positive staining, indicating that the tumor cells were actively dividing. (F) TRPS1 staining is commonly used for breast cancer. Large nuclei were observed in areas of high staining, large nuclei are observed, and the deep staining suggested the presence of metastatic tumor cells.

**Figure 2.**
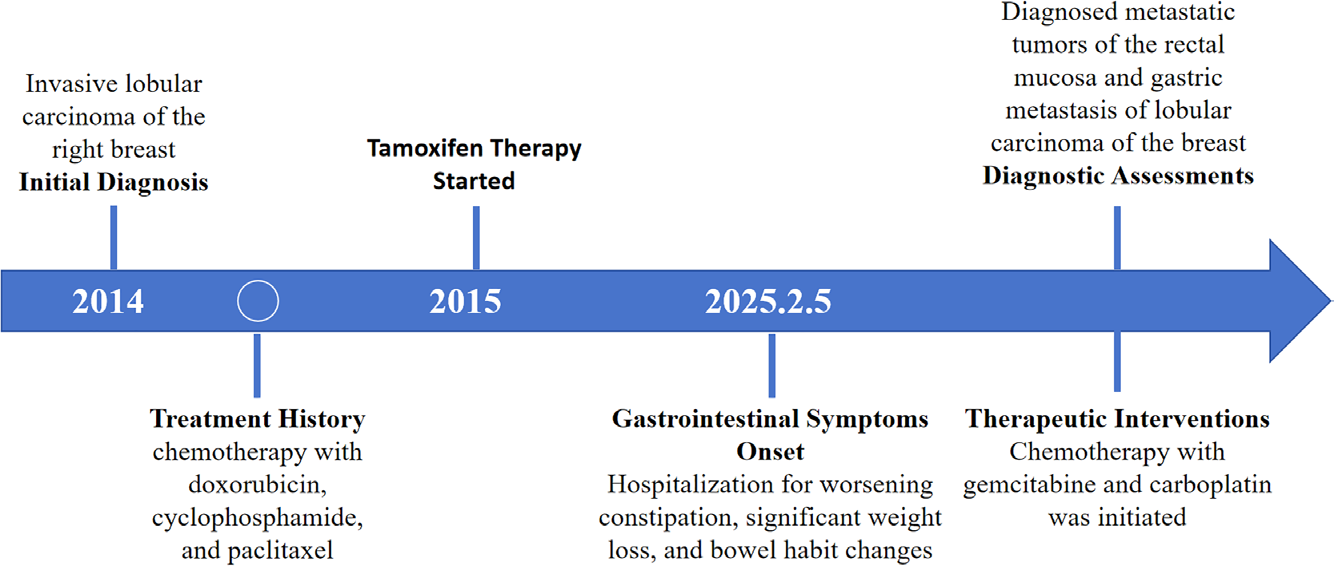
Timeline of clinical course.

A 49-year-old woman was diagnosed with invasive lobular carcinoma of the right breast in 2014 and subsequently underwent a modified radical mastectomy. She received 8 cycles of doxorubicin and cyclophosphamide, followed by sequential paclitaxel, and was started on tamoxifen in 2015. Routine follow-ups from 2014 to 2025 revealed no evidence of recurrence or metastasis. On February 5, 2025, the patient was hospitalized with a 6-month history of worsening constipation, significant weight loss, and altered bowel habits. Abdominal examination was unremarkable, and digital rectal examination revealed no palpable masses. Tumor markers revealed a carcinoembryonic antigen level of 5.01 ng/mL and a carbohydrate antigen 724 level of 64.32 U/mL, with no other significant abnormalities. Abdominal computed tomography (CT) showed thickening of the gastric antrum and rectal wall, resulting in luminal narrowing. Flexible colonoscopy identified small fragments of mucosal tissue 20 cm from the anal verge, showing mild edema, lymphocytic infiltration, and small vessel wall thickening, which caused luminal narrowing or obstruction. The rectal mucosa exhibited chronic inflammation, focal lymphocytic aggregation, and occasional signet-ring cell-like cells. Immunohistochemistry demonstrated positivity for CK, CD68, Ki-67, and TRPS1. Upper endoscopy revealed a polyp in the gastric cardia, with the lamina propria of the angularis containing solitary or clustered round epithelial-like cells. Immunohistochemical analysis showed positivity for TRPS1, P120, and GATA-3; negativity for CD68 and E-cadherin; weak-to-moderate estrogen receptor positivity (70%); weak progesterone receptor positivity (1%); Ki-67 positivity (3%); and HER2 score of 1+ (Fig. 1). The clinical and pathological findings supported a diagnosis of metastatic lobular carcinoma involving the rectal mucosa and stomach. Following multidisciplinary evaluation, the patient began chemotherapy with gemcitabine and carboplatin. Three cycles of chemotherapy were scheduled, followed by repeat colonoscopy to assess treatment response and ongoing surveillance for disease progression. The patient’s clinical course is summarized in Figure 2.

## 3. Discussion

Gastrointestinal metastases developed 11 years after the patient underwent surgery for ILC, presenting as 6 months of bowel movement difficulty with recent symptom worsening. Abdominal CT revealed thickening of the gastric antrum and rectal wall, accompanied by luminal narrowing. Endoscopic biopsy confirmed metastatic breast cancer in the rectal mucosa, while the stomach demonstrated metastatic lobular carcinoma. These findings are consistent with the characteristic features of gastrointestinal metastases from breast cancer, typically presenting as thickening and narrowing of the gastrointestinal wall.^[8]^ Due to the nonspecific clinical presentation of gastrointestinal involvement, histopathological and immunohistochemical analyses are essential for diagnosis. Histological evaluation revealed changes in the intestinal mucosa: the colonic mucosa, located 20 cm from the anal verge, exhibited mild edema, lymphocytic infiltration, and small vessel wall thickening, resulting in luminal narrowing; the rectal mucosa showed features of chronic inflammation. Immunohistochemistry revealed positivity for CK and CD68, scattered Ki-67-positive cells, and TRPS1 positivity, indicating tumor cell infiltration.

Upper endoscopy revealed a polyp in the gastric cardia, with solitary or clustered round epithelial-like cells observed in the lamina propria of the angularis. Immunohistochemical analysis showed positivity for TRPS1, P120, and GATA-3, and negativity for CD68 and E-cadherin. TRPS1, a transcriptional repressor belonging to the GATA family, has emerged as a highly sensitive and specific marker for breast carcinoma, particularly ILC.^[9]^ Studies have demonstrated that TRPS1 is expressed in the majority of breast cancers but rarely in primary gastric or colorectal adenocarcinomas.^[10,11]^ Similarly, GATA-3 is a well-established marker for breast epithelium and is frequently expressed in both ductal and lobular breast carcinomas, with limited expression in gastrointestinal tumors.^[12]^ In contrast, E-cadherin loss is a characteristic feature of ILC due to CDH1 gene inactivation, and its absence in metastatic lesions supports a lobular origin.^[13]^ The negative expression of E-cadherin and CD68, coupled with TRPS1 and GATA-3 positivity, thus provides strong immunophenotypic evidence favoring breast origin over a primary GI source. The estrogen receptor was weakly to moderately positive, the progesterone receptor was weakly positive, Ki-67 was positive, and HER2 was scored as 1+. When these findings are considered alongside the immunohistochemical profile of the patient’s primary breast cancer in 2014—estrogen receptor positive in 50%, progesterone receptor positive in 80%, HER2 2+, Ki-67 5%, CK positive, p53 3%, and cytoplasmic p120 positive—it can be inferred that the rectal lesions are consistent with metastatic breast cancer, while the gastric lesions are associated with metastatic lobular carcinoma, supporting the diagnosis of secondary breast cancer.

Although gastrointestinal metastases from ILC have been increasingly recognized, this case is notable for several rare features. First, the interval between initial treatment and gastrointestinal recurrence was 11 years, which exceeds the commonly reported median disease-free interval of approximately 6 years in most large case series.^[14,15]^ Delayed recurrence after a decade or more is seldom documented, highlighting the importance of long-term vigilance in ILC patients. Second, concurrent metastases to both the stomach and rectum are exceedingly uncommon. Most published cases involve single-site gastrointestinal involvement, typically in the stomach, small intestine, or colon.^[16,17]^ A dual-site presentation in the upper and lower gastrointestinal tract, as observed in this patient, suggests a broader pattern of dissemination and may pose greater diagnostic challenges due to overlapping or nonspecific symptoms. Taken together, this case adds to the limited literature on long-latency, multi-site gastrointestinal metastases from ILC and underscores the critical role of immunohistochemistry in distinguishing such lesions from primary GI malignancies.

Treatment strategies for gastrointestinal metastases differ significantly from those for primary gastrointestinal cancers, underscoring the importance of an accurate diagnosis. The most common manifestation of breast cancer metastasis to the stomach is infiltrative lesions.^[18]^ Immunohistochemical analysis is essential for distinguishing metastatic from primary gastric cancers. Chemotherapy or radiotherapy is typically used to treat metastatic gastrointestinal tumors, while surgery is generally reserved for patients with complications.^[19]^ The selection of systemic therapy depends on patient age, presenting symptoms, functional status, estrogen receptor status, and prior treatments. The median survival of patients with breast cancer metastases to the gastrointestinal tract ranges from 10 to 28 months.^[14,19]^ However, studies have shown that patients with invasive lobular carcinoma exhibit lower chemotherapy response rates; thus, this should be carefully considered when selecting an optimal treatment regimen.^[20]^

Given the indolent nature of invasive lobular carcinoma and its potential for late recurrence, a structured follow-up strategy is essential. In this case, the patient is scheduled to undergo follow-up flexible colonoscopy and upper endoscopy after completion of 3 cycles of chemotherapy, in order to evaluate mucosal response and residual disease. In addition, cross-sectional imaging with contrast-enhanced CT or MRI will be performed every 3 to 6 months to monitor for disease progression or emergence of new metastatic lesions. Serum tumor markers (e.g., CEA, CA 72-4) will also be reassessed regularly as part of ongoing surveillance. Given the weak-to-moderate estrogen receptor positivity in metastatic lesions, reevaluation of endocrine therapy options, including switching to aromatase inhibitors or adding CDK4/6 inhibitors, will be considered after systemic therapy stabilization. Long-term follow-up will focus on maintaining quality of life, monitoring treatment tolerance, and detecting late complications or recurrence at extrapulmonary sites.

Advances in tumor immunology and molecular profiling have provided critical insights into the long-term behavior of breast cancer, particularly ILC. For example, a recent study demonstrated that systemic inflammatory markers, such as neutrophil-to-lymphocyte ratio and platelet-based indices, are associated with poorer prognosis and may guide posttreatment surveillance in patients with breast cancer.^[21]^ These findings underscore the importance of incorporating host inflammatory status into long-term prognostic stratification. Moreover, emerging single-cell transcriptomic analyses have identified novel immune-regulatory markers associated with breast cancer progression. IL27RA and TMEM71, for instance, have been identified as subtype-specific immune markers in ILC and other breast cancer subtypes, potentially influencing metastatic tropism and treatment responsiveness.^[22,23]^ These markers may help explain the distinct immunologic microenvironment observed in gastrointestinal metastases and point to potential avenues for immuno-targeted therapies. Notably, the prognostic value of inflammation-based indices is not restricted to breast cancer. A parallel study in colorectal cancer demonstrated that systemic immune-inflammation markers correlate with tumor progression and recurrence risk.^[24]^ Analogous inflammation-driven mechanisms observed in gastrointestinal malignancies further underscore the role of immune contexture in breast cancer metastasis, particularly to gastrointestinal sites. Collectively, these findings support incorporating immune and inflammation-based biomarkers into future prognostic models and surveillance strategies, particularly in patients with atypical metastatic patterns and prolonged latency, as illustrated in the present case.

## 4. Conclusion

In patients with a history of breast cancer who develop new gastrointestinal symptoms or are diagnosed with primary gastric or colorectal cancer, clinicians should maintain a high index of suspicion for metastatic disease. Histopathological and immunohistochemical findings from biopsy specimens should be thoroughly evaluated and compared with the original breast cancer profile to ensure diagnostic accuracy. In this case, the patient presented with nonspecific gastrointestinal symptoms many years after being diagnosed with invasive lobular carcinoma. Clinicians should be particularly vigilant in patients with positive fecal occult blood tests and consider further investigations to rule out metastases, especially in cases involving large tumors or lymph node involvement.

## Acknowledgments

We thank the patient, for agreeing to the publication of his images and clinical information.

## Author contributions

**Conceptualization:** Haiyuan Yang, Lichao Zhu.

**Data curation:** Yiheng Zhang, Guoxin Sun.

**Funding acquisition:** Yating Zhao.

**Writing – original draft:** Jie Peng.

**Writing – review & editing:** Ye Liu.
